# Overcoming tumor resistance mechanisms in CAR-NK cell therapy

**DOI:** 10.3389/fimmu.2022.953849

**Published:** 2022-08-03

**Authors:** Antonio Valeri, Almudena García-Ortiz, Eva Castellano, Laura Córdoba, Elena Maroto-Martín, Jessica Encinas, Alejandra Leivas, Paula Río, Joaquín Martínez-López

**Affiliations:** ^1^ Hospital Universitario 12 de Octubre-Centro Nacional de Investigaciones Oncológicas (H12O-CNIO) Haematological Malignancies Clinical Research Unit, Spanish National Cancer Research Centre, Madrid, Spain; ^2^ Department of Hematology, Hospital Universitario 12 de Octubre-Universidad Complutense, Instituto de Investigación Sanitaria Hospital 12 de Octubre (imas12), Madrid, Spain; ^3^ Division of Hematopoietic Innovative Therapies, Biomedical Innovation Unit, Centro de Investigaciones Energéticas Medioambientales y Tecnológicas (CIEMAT), Centro de Investigación Biomédica en Red de Enfermedades Raras (CIBERER) and Instituto de Investigación Sanitaria Fundación Jiménez Díaz, Universidad Autónoma de Madrid (IIS-FJD, UAM), Madrid, Spain

**Keywords:** chimeric antigen receptor (CAR), CAR NK cells, hematologic tumor, genome editing, CRISPR/Cas9, tumor microenvironment, tumor resistance, CAR persistence

## Abstract

Despite the impressive results of autologous CAR-T cell therapy in refractory B lymphoproliferative diseases, CAR-NK immunotherapy emerges as a safer, faster, and cost-effective approach with no signs of severe toxicities as described for CAR-T cells. Permanently scrutinized for its efficacy, recent promising data in CAR-NK clinical trials point out the achievement of deep, high-quality responses, thus confirming its potential clinical use. Although CAR-NK cell therapy is not significantly affected by the loss or downregulation of its CAR tumor target, as in the case of CAR-T cell, a plethora of common additional tumor intrinsic or extrinsic mechanisms that could also disable NK cell function have been described. Therefore, considering lessons learned from CAR-T cell therapy, the emergence of CAR-NK cell therapy resistance can also be envisioned. In this review we highlight the processes that could be involved in its development, focusing on cytokine addiction and potential fratricide during manufacturing, poor tumor trafficking, exhaustion within the tumor microenvironment (TME), and NK cell short *in vivo* persistence on account of the limited expansion, replicative senescence, and rejection by patient’s immune system after lymphodepletion recovery. Finally, we outline new actively explored alternatives to overcome these resistance mechanisms, with a special emphasis on CRISPR/Cas9 mediated genetic engineering approaches, a promising platform to optimize CAR-NK cell function to eradicate refractory cancers.

## Introduction

Over the last decade, autologous CAR-T therapy has revolutionized the treatment of hematological tumors as reflected in six different CAR-T treatments that have received marketing authorization so far to treat multiple myeloma (MM) and CD19^+^ B cell malignancies, and are now routinely used in the clinic (1–7). Despite their undoubtedly clinical success in the relapsed and refractory setting, CAR-T real-world clinical experience reveals challenges such as cumbersome manufacturing and high-grade toxicities (8) as well as sub-optimal long-term disease control for many patients (1, 3, 9), associated with different mechanisms of resistance that have been extensively reviewed in Shah et al. (10) and in this article collection. Moreover, outcomes for patients who finally progress after CAR-T cell therapy are dismal (11). These limitations highlight the need to investigate alternative immune effector cells as potential vehicles for CAR engineering.

**Figure 1:**
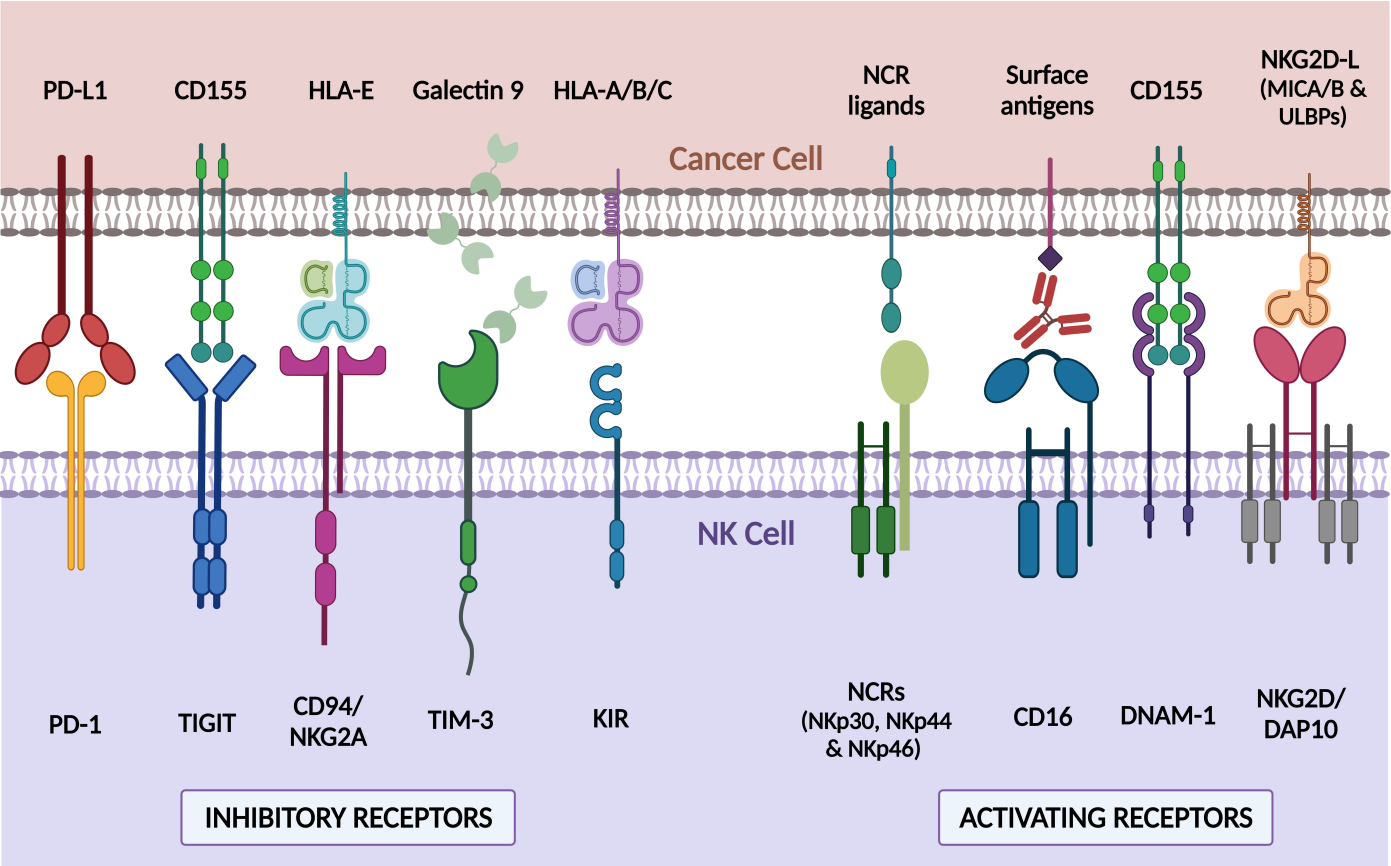
Major endogenous NK cell receptors and their associated ligands in tumor cells. NK cell function is modulated through different surface receptors which bind to ligands expressed on cancer cells. Receptors such as NCR (NKp30, NKp44, and NKp46), CD16, DNAM-1, or NKG2D/DAP10 trigger an activating NK signaling that results in a potent cytotoxic response against ligand-expressing cells. On the contrary, other receptors like PD-1, TIGIT, CD94/NKG2A, TIM-3, and KIRs, turn off NK response when bind to their cognate ligands. The combination of positive and negative signals regulates NK cell response to target cells. PD-1, Programmed Death 1; TIGIT, T cell immunoglobulin and ITIM domain; TIM-3, T cell immunoglobulin and mucin-domain containing-3; KIR, Killer-cell immunoglobulin-like receptor; NCRs, natural cytotoxicity receptors; DNAM-1, DNAX accessory molecule; NKG2D/DAP10, natural killer group 2D/DNAX-activation protein 10; PD-L1, Programmed Death ligand-1; HLA-E, HLA class I histocompatibility antigen, alpha chain E; HLA-A/B/C, HLA class I histocompatibility antigen, alpha chain A/B/C; NKG2D-L, NKG2D ligands; MICA/B, MHC class I polypeptide-related sequence A/B; ULBPs, UL16 binding proteins. Created with BioRender.com.

CAR-NK cells emerge as strong candidates due to the unique biological properties and multiple mechanisms of action of conventional Natural Killer (NK) cells. NK cells are innate effector lymphocytes but can also exhibit features of memory-like or adaptive response (12–14). The main function of NK cells is to identify and rapidly discriminate and kill virally infected, stressed, or senescent cells and control several types of tumor cells and metastases (15–17). Human NK cells have been traditionally subclassified into immature immunomodulatory NK cells (CD56^bright^CD16^-/dim^) and the mature NK cell (CD56^dim^CD16^bright^) subset, which mediates the cytolytic function (18, 19). In contrast to T cells, adoptive NK or CAR-NK therapy does not cause serious adverse events, such as on-target off-tumor toxicities, cytokine release syndrome (CRS), or immune effector cell‐associated neurotoxicity syndrome (ICANS), which may increase hospitalization length and raise therapy cost (20–22). The short NK lifespan *in vivo* and the different spectrum of cytokines and growth factors released during NK cell killing (e.g, TNF-α, IFN-γ, GM-CSF, and IL-3), are probably responsible for these advantages (23, 24). Allogeneic NK products surpass the expensive and lengthy procedure of autologous CAR-T manufacturing (25). Besides, they are also exempt from *ex vivo* expansion failures reported in heavily pre-treated patients (10-30%) (1) and tumor contamination events occurring during autologous CAR-T cell productions (26). Allogenic NK and CAR-NK cells constitute an “off‐the‐shelf” product for immunotherapy that can be applied to different patients and generated from multiple sources (20–22, 27). This potential arises due to their minimal risk to cause graft‐versus‐host disease (GvHD). NK cells are functionally similar to CD8^+^ T cells but do not require prior sensitization and lack TCR expression thereby their responses are not human leukocyte antigen (HLA)-restricted. Instead, NK cell function depends on the balance between activating and inhibitory signaling generated by several germline‐encoded receptors (see review in Sivore et al. and Xie et al.) (**Figure 1**) (28, 29). Thus, NK cells retain CAR-independent killing capacity through these innate receptors even in a tumor escape scenario characterized by CAR antigen loss or down-regulation. NK cells could eliminate tumor cells through CD16-mediated antibody-dependent cell-mediated cytotoxicity (ADCC), direct target killing by cytolytic granules, (e.g. perforin and granzymes), or *via* engagement of death receptors (e.g. FASL or TRAIL) (30). Additionally, NK cells efficiently produce cytokines and chemokines that modulate other immune mediators of cytotoxicity (31). Therefore, re-directing NK cells to express a CAR potentially synergizes to kill heterogeneous tumors and reduce the risk of relapse due to CAR-dependent mechanisms.

A large number of CAR-NK preclinical studies have been revealed to be effective in cancer therapy, particularly in the treatment of hematological malignancies (see Gong et al. and Daher et al., for exhaustive review) (32, 33). Up to now, 31 clinical trials are registered with 11 different CAR targets (CD19, CD20, CD22, NKG2D-L, CD33, and BCMA on the top) to address the clinical efficacy of CAR-NK cells in hematologic tumors (**Table 1**) (22, 34). The most promising data reported arise from first-in-human phase I/II CAR-NK clinical trials based on primary umbilical cord blood (UCB) CAR-NK and induced pluripotent stem cell (iPSC)-derived CAR-NK cell products (35). Rapid and impressive responses (ORR: 73% CR: 64%) were achieved with a bicistronic CD19-28-ζ CAR/IL-15 UCB NK cells in chronic lymphocytic leukemia (CLL) and lymphoma refractory and relapsed setting (NCT03056339). However, the durability of the response in this study could not be completely assessed in some patients because other therapies were administered 30 days after the infusion of CAR-NK cells (22, 36). In the same way, interim analysis in the single-dose cohort treated with FT596, a multi-engineered effector generated using a construct containing a CD19-targeting CAR, a high-affinity uncleavable CD16 (hnCD16) Fc receptor, and an IL-15/IL-15R fusion, revealed an estimated ORR of 69% and CR of 56% in combination with an anti-CD20 antibody (37). Recently, new data from a few patients have been released from NKX-101 (NKG2D CAR-NK in acute myeloid leukemia (AML) and myelodysplastic syndrome (MDS)) (3/5 patients achieved CR) and NKX-019 (CD19 CAR-NK in CD19^+^ B acute lymphocytic leukemia (ALL) and lymphoma) (5/6 patients in CR) (preliminary dose-finding data report, Nkarta, April 2022). While we look forward to seeing efficacy confirmation in the interim analysis of related ongoing products, such as TK-007 (CD19-28-ζ CAR/IL-15 UCB NK cells) or FT576 (iPSC-derived BCMA CAR-NK in MM), these early results suggest similar high-quality responses using CAR-NK cells as compared to CAR-T cells. Importantly, in contrast to CAR-T cell therapy, no evidence of severe CRS, ICANS, hemophagocytic lymphohistiocytosis (HLH), or life-threatening GvHD was observed in any of the aforementioned trials using CAR-NK cells.

**Table 1:** Current CAR-NK therapy clinical trials in hematological tumors.

Row	Status	Disease	Construct/Combination	NK Cell sources	Phase	N^ar^ patients	Country	NCT number
1	Recruiting	Acute Myeloid Leukemia	NKG2D CAR	Cord blood	Phase 1	9	China	NCT05247957
2	Recruiting	B-cell Non Hodgkin Lymphoma	CD19 CAR	Haploidentical donor	Phase 1	25	China	NCT04887012
3	Recruiting	Acute Myeloid Leukemia	CD33/CLL1 CAR	Unknown	Early Phase 1	18	China	NCT05215015
4	Not yet recruiting	Non Hodgkin Lymphoma	CD19 CAR	Unknown	Early Phase 1	9	China	NCT04639739
5	Recruiting	Acute Myeloid Leukemia	CD33 CAR	Unknown	Phase 1	27	China	NCT05008575
6	Unknown	Refractory B-Cell Lymphoma	CD22 CAR	Unknown	Early Phase 1	9	Unknown	NCT03692767
7	Unknown	Refractory B-Cell Lymphoma	CD19 CAR	Unknown	Early Phase 1	9	Unknown	NCT03690310
8	Recruiting	Refractory Multiple Myeloma	BCMA CAR	Cord blood	Early Phase 1	27	China	NCT05008536
9	Recruiting	Multiple Myeloma	BCMA CAR	NK-92 cell line	Phase 1/2	20	China	NCT03940833
10	Unknown	Refractory B-cell Lymphoma	CD19/CD22 CAR	Unknown	Early Phase 1	10	Unknown	NCT03824964
11	Recruiting	Relapsed/Refractory Acute Myelodysplastic Syndromes	NKX101 (NKG2D CAR/mbIL-15)	Haploidentical donor (PB)	Phase 1	90	USA	NCT04623944
12	Recruiting	B-cell Leukemias Waldenstrom Macroglobulinemia B-cell Lymphomas	NKX019 (CD19 CAR/mbIL-15)	Donor (PB)	Phase 1	60	USA Australia	NCT05020678
13	Unknown	Acute Myeloid Leukemia Acute nonlymphocytic leukemia	CD33 CAR	NK-92 cell line	Phase 1/2	10	China	NCT02944162
14	Recruiting	Acute Lymphocytic Leukemia Chronic Lymphocytic Leukemia Non Hodgkin’s Lymphoma	CD19 CAR	Cord blood	Phase 1	27	China	NCT04796675
15	Unknown	B-cell Leukemias B-cell Lymphomas	CD19 CAR	NK-92 cell line	Phase 1/2	10	China	NCT02892695
16	Withdrawn	B-cell Lymphomas	CAR CD19/iCasp9/IL 15 + Rituximab	Cord blood	Phase 1/2	0	USA	NCT03579927
17	Active not recruiting	B-cell Leukemias B-cell Lymphomas	iCasp9/CAR CD 19/IL15 + AP1903	Cord blood	Phase 1/2	36	USA	NCT03056339
18	Recruiting	Multiple Myeloma	FT576(BCMA CAR) + Daratumumab	iPSCs	Phase 1	168	USA	NCT05182073
19	Not yet recruiting	B-cell Lymphoma Myelodysplastic Syndromes Acute Myeloid Leukemia	CARCD70/IL 15	Cord blood	Phase 1/2	94	USA	NCT05092451
20	Not yet recruiting	CD19-Positive B-cell Malignancies	CNTY-101 (CAR CD19/EGFR/IL15) +IL-2	iPSCs	Phase 1	75	Unknown	NCT05336409
21	Not yet recruiting	Hematological Malignancies	CAR CD5/IL 15	Cord blood	Phase 1/2	48	USA	NCT05110742
22	Unknown	CD7-Positive Leukemia or Lymphoma	CD7 CAR	NK-92 cell line	Phase 1/2	10	China	NCT02742727
23	Recruiting	B-cell Lymphoma	FT596 (CD19 CAR)	iPSCs	Phase 1	50	USA	NCT04555811
24	Recruiting	B-cell Lymphoma Chronic Lymphocytic Leukemia	FT596 (CD19 CAR) + Rituximab	iPSCs	Phase 1	285	USA	NCT04245722
25	Completed	Acute Lymphoblastic Leukemia	CD19 CAR	Haploidentical donor (PB)	Phase 1	14	USA	NCT00995137
26	Withdrawn	Diffuse Large B Cell Lymphoma	CD19 CAR	Modified NK-92 (haNK)	Phase 1	0	USA	NCT04052061
27	Suspended	B-cell Acute Lymphoblastic Leukemia	CD19 CAR + IL-2	Haploidentical donor (PB)	Phase 1	20	Singapore	NCT01974479
28	Unknown	B-cell Leukemias B-cell Lymphomas	CD19 CAR	NK-92 cell line	Phase 1/2	10	China	NCT02892695
29	Recruiting	B-cell Non Hodgkin Lymphoma	CAR CD19/IL15	Cord blood	Phase 2	242	USA	NCT05020015
30	Recruiting	B-cell Acute Lymphoblastic Leukemia (B-ALL) B-cell Lymphoma	QN-019a (CAR CD19) +/- Rituximab	Allogenic	Phase 1	24	China	NCT05379647
31	Recruiting	Acute Lymphocytic Leukemia, Chronic Lymphocytic Leukemia, Non Hodgkin Lymphoma	CD19 CAR	Unknown	Phase 1	15	China	NCT05410041

Despite the multiple advantages, CAR-NK therapy still has to confront additional shortcomings that provoke resistance and impact on its efficacy, as seen in CAR-T cells (38). Herein, we will discuss these mechanisms, especially focusing on the CAR-NK dysfunctionalities that lead to immune surveillance evasion by hematologic tumors. We will also review current strategies, mainly based on genome editing, to overcome CAR-NK functional exhaustion and limited migration, and harness CAR-NK effectors replication and persistence *in vivo*.

## Manufacturing conditions: Key aspect for the efficacy of CAR-NK therapy

One of the main concerns regarding adoptive cell therapy is the need for a great number of enhanced functional effector cells with potential proliferative capacity for optimal clinical responses. Thus, optimizing the source, the cell cytokine-priming, and the expansion protocols can determine CAR-NK cell cytotoxicity and maintenance *in vivo*.

### Cytokine priming and expansion methods

The most common sources used as platforms to develop CAR-NK therapy are NK cells from peripheral blood (PB) or UCB, NK cell lines (such as NK92), and stem cell-derived NK cells, generated from iPSCs, human embryonic stem cells (hESCs) or CD34^+^ hematopoietic stem cells (39). PB-NK cells are mature cells with high cytotoxic activity and extended expansion potential but show challenges regarding genetic modification. In contrast, UCB-NK cells contain mainly immature NK cells that after an expansion process acquire a cytotoxic status with equivalent functionality to PB-NK cells (40). Due to donor variability, both PB and UCB-NK cells are heterogeneous products. NK92 cell line provides unlimited homogeneous effectors with easy manufacturing expansion and genetic manipulation but mandatory irradiation before infusion for safety concerns impedes their persistence. In fact, CD33-CAR NK-92 cells do not appear to be effective against AML in the first CAR-NK92 clinical trial (NCT02944162) (34). Alternatively, iPSC-derived NK cells are a homogeneous, unlimited, and easy-to-edit option but their manufacturing is lengthy and requires specialized expertise (41).

Given their clinical suitability and that GvHD was not expected, first approaches for NK adoptive cell therapy were performed with autologous PB-NK cells. In spite of the proven safety, no clinical efficacy was observed due to self-tolerance mediated by HLA-matching (42, 43). Besides, NK cell repertoire in hematological malignancies patients is reduced and functionally altered by the tumor and previous aggressive treatments (44). Pioneer studies from Ruggeri et al. reported that host HLA-I-donor KIR mismatch can promote NK cell graft-versus-leukemia effect (GvL) in transplant setting (“missing-self”), therefore allogenic NK cells have been preferentially selected because exhibit additional advantages to being ready-to-use product (27). Since then, many studies have been focused on haploidentical NK therapy also in non-transplant context, demonstrating safety and modest clinical responses (45). Nevertheless, autologous NK cell immunotherapy is re-emerging in MM clinical consolidation setting with promising data of efficacy released (46). Taken together, these findings highlight that NK cell sources can impact adoptive NK cell therapy clinical outcomes and that the addition of a suitable CAR could unleash NK cell functionality even in inhibitory KIR/HLA-I compatible settings (“induced-self”).

Regarding NK cell priming and expansion strategies, most of them are based on the use of soluble cytokines and artificial antigen-presenting cells (aAPC) with membrane-bound molecules such as cytokines and/or costimulatory ligands (in-depth reviewed by Gurney et al. and Liu et al. (47, 48)). Common gamma-chain cytokines IL-2, IL-7, IL-15, and IL-21, and others like IL-12 or IL-18, alone or in combinations are the most commonly studied (49–51). The use of irradiated feeder cells like K562 genetically modified to express membrane-bound IL-15 or IL-21 (mbIL-15/mbIL-21) and 4-1BBL greatly increases fold-expansion rates while maintaining the cytotoxic potential of NK and CAR-NK cells (21, 22, 52–55). Other presenting cells such as Epstein-Barr Virus transformed Lymphoblastoid Cell Lines (EBV-LCLs) have also been studied. Yang et al. engineered a human B-lymphoblastoid cell line with mbIL-21 that provided higher NK cell expansion compared to conventional K562.mbIL-21 and a more favorable phenotype regarding functionality and proliferative capacity (56). aAPCs have demonstrated to efficiently expand NK cells and to be clinically safe (57), but cell-free approaches such as liposomal particles with mbIL-21 and 4-1BBL (58), membrane patches from K562.mbIL21.41BBL (59) or hyaluronic acid-based biomaterials (60) are also under investigation.

Despite some authors suggest optimal landscapes to boost NK cell proliferation (61), a harmonization between NK/CAR-NK expansion protocols is yet to be established. Cytokines such as IL-2, IL-15, and IL-21 play key roles in NK cell functionality and development (49, 62, 63), thus their exposure should be exhaustively addressed as may critically contribute to product efficacy. NK cells can become “addicted” to supraphysiological cytokine exposure, suffering a dramatic drop after interleukin withdrawal when infused into patients, limiting their persistence and efficacy *in vivo* (64). Molecular mechanisms leading to NK cell decline in the absence of interleukin stimulus are caspase 3 activation, decrease in BCL-2/BIM ratio, and induction of a proapoptotic splice variant of BIM (65). Consequently, *in vivo* administration of these cytokines was proposed to circumvent *ex vivo* signaling dependence.

Implications of systemic IL-2 supply to potentiate NK cell expansion were evaluated by Miller’s lab. The infusion of haploidentical NK cells with concomitant IL-2 support after a lymphodepleting chemotherapy obtained disappointing results as NK cell growth was inhibited by host regulatory T cells (Tregs) given that their IL-2Rα provides them with a higher affinity for IL-2 (66). Hence, they depleted Tregs with IL-2-diphtheria toxin fusion protein, prompting NK cell expansion immediately after lymphodepletion, achieving heighten CR rates (NCT00274846 and NCT01106950) (67). Systemic IL-2 administration has related toxicities such as capillary leak syndrome (68, 69), arising the need for using other cytokines with high NK selectivity, such as IL-15.

IL-15 shares similarities with IL-2 but has a high affinity for its IL-15Rα, thus stimulating NK cells but not Tregs (70). The short half-life of IL-15 (71) has promoted the development of alternative related molecules to overcome this drawback. Clinical Trials using either rhIL-15 (72) or IL-15 engineered molecules (N-803, formerly known as ALT-803 (NCT01885897, NCT02384954) (73–75) and NKTR-255 (76, 77)) demonstrated great NK and CD8^+^ T cell expansion and minimal effect over CD4^+^ T cells or Tregs but performed insufficient potency themselves (78–80). Moreover, Cooley et al. described CRS in around half of the patients receiving subcutaneous (but not intravenous) IL-15, who also had high IL-6 levels, suggesting that IL-15 stores could trigger proinflammatory cytokines release by myeloid cells (81). Other undesired effects such as neutropenia in nonhuman primates (82) or leukemia in mice (83, 84) have been associated with rhIL-15 systemic administration.

IL-21 is another common gamma-chain cytokine that has demonstrated biasing NK cells for a mature functional phenotype with augmented granule release (85–87), heightened IFN-γ secretion (88), and manageable toxicity in phase I-II clinical trials (89). Still, its effects seem to depend on exposure conditions (90) and high IL-21 has been related to apoptosis *in vitro* (85). Hence, its systemic supply should be accurately controlled.

As systemic administration of cytokines themselves also entails undesired effects, the newest engineering approaches are focused on *in situ* delivery and harnessing cytokine signalization to prolong NK/CAR-NK cell persistence while maintaining their optimized functionality. For instance, Liu et al. developed an IL-15 autocrine secreting CD19 CAR UCB-NK cell that showed enhanced cytotoxicity *in vitro* (91) and CAR-NK cell persistence in a phase I/II clinical trial without systemic IL-15 level increase in the patients (NCT03579927) (22). To further improve their candidate, they ablated CISH, a gene that encodes the cytokine checkpoint CIS (Cytokine inducible Src homology 2 containing protein), which restrains IL-2 and IL-15 signaling (92), obtaining optimal proliferation rates by increasing CAR-NK cell metabolic fitness *via* glycolysis potentiation (93). Zhu et al. also demonstrated benefits in persistence and antitumor effect of CISH-depleted iPSC NK cells in an AML mouse model (94). Cytokine signalization components have been modified to boost CAR-NK activity by incorporating the inducible MyD88/CD40 (iMC) system as an independent co-stimulator of an IL-15 secreting CAR-NK to enhance cell persistence and tumor control (95, 96). Other IL-15 armored CAR-NK cells developed by Zicheng Du et al. (97) and Christodoulou et al. (98) showed the same trend for CAR-NK cell persistence but in the latter, much lesser potency was achieved and dramatic systemic toxicity was observed. Therefore, other cytokine supply methods are being evaluated.

For instance, engineering NK cells to express mbIL-15 may provide benefits averting the aforementioned undesired effects of systemic IL-15. Additionally, mbIL-15 has demonstrated higher functionality in mice compared to the soluble form even at physiological levels (99). Imamura et al. developed mbIL-15 human NK cells with autonomous potential growth, activated antiapoptotic pathways, and enhanced antitumor effect toward hematological and solid cancers both *in vitro* and *in vivo* (100). Going a step beyond, CD19 CAR-NK cells have been engineered to express IL-15/IL-15 receptor α (IL-15/IL-15Rα) fusion protein, endowing them with enhanced persistence regarding their IL-15 secreting counterpart and potentially sustaining tumor control (101). Similarly, IL-15/IL-15Rα has been included in iPSC-derived CAR-NK cells against MM (FT576) (102) or B cell malignancies (FT596) (103), being the latter under assessment in a phase I clinical trial (NCT04245722) (37).

Differentiation into a memory-like setting is a unique strategy to enhance *in vivo* expansion, persistence, and antitumor responses. Romee, Fehniger, and colleagues demonstrated that brief priming with an IL-12, IL-15, and IL-18 cocktail reprogrammed allogeneic NK cells to a cytokine-induced memory-like (CIML) phenotype that endowed them with heightened expansion and persistence *in vivo*, higher interferon-γ (IFN-γ) production, and enhanced cytotoxicity against AML and other malignancies (13, 104–106). These acquired advantages have been reproduced in different preclinical studies and phase I/II clinical trials demonstrating a suitable safety profile and promising efficacy, achieving 56% OR rate and 44% CR rate in AML and MDS patients (NCT01898793). CIML-NK clinical trials are also ongoing in a haploidentical hematopoietic cell transplantation (HCT) context (NCT02782546) (107) or donor-derived adoptive therapy in post-HCT AML relapse setting (NCT04024761, NCT03068819) (108, 109). Moreover, CIML-NK combinatorial approaches with chemotherapy (e.g. Ara-C), IL-15 superagonist (NCT02782546) (107), NK-cell engagers (NCT04074746) (110), or a CD38-antibody recruiting molecule (NCT04634435) (111) are also being developed. Regarding CAR-NK context, CIML-NK modified to express CD19 CAR exhibited synergism on CAR activation and demonstrated the aforementioned CIML-NK advantages in an NK-resistant lymphoma model (112). Very recently, Romee’s lab showed that arming CIML NK cells with TCR-like CAR against intracellular neoepitope nucleophosphmin-1 (NPM1) improves anti-AML responses and could be considered as a treatment for NPM1c-mutated HLA-A2^+^ AML patients (113).

### Fratricide diminishes NK cell efficacy

NK cell *ex vivo* expansion can entail an undesired phenomenon known as fratricide, by which cells recognize receptors or ligands on the surface of their siblings and trigger a cytotoxic activity against them. Several mechanisms can lead to fratricide during NK or CAR-NK cell expansion.

Among them, the well-known Fas/FasL axis is one of the most relevant mechanisms. FasL-mediated cytotoxicity plays a key role in NK cell functionality since it triggers caspase-dependent apoptosis when binds to its receptor Fas in target cells. Fas can also be physiologically expressed by NK cells as a homeostatic mechanism to restrain NK cell activity, termed activation-induced cell death (AICD), but it has been reported that its expression can be abnormally increased during NK cell expansion, especially when cultured in the presence of IL-2 (114), continuous IL-15 treatment (115) or specific feeder cells such as K562-mIL21 (56), leading to fratricide. Moreover, apart from a “self-killing” effect, the enhanced expression of Fas during CAR-NK cell expansion concurrently with a FasL overexpression that has been described in tumor cells such as malignant plasma cells (116) or in the tumor microenvironment (TME) (117), may contribute to tumor escape to adoptive cell therapy.

Another receptor potentially causing fratricide among NK cells is NKG2D. NKG2D is a natural receptor mainly expressed by NK, CD8^+^ T, and γδ T cells that recognizes several stress-induced ligands (NKG2D-Ls; MHC class I chain-related molecule A/B (MICA/B) and UL16-binding protein (ULBP) 1-6), frequently expressed by cancerous or virally infected cells. There are increasing data describing NKG2D-L expression by activated NK cells but its origin and implications regarding NK cell functionality remain controversial (118). Some studies described that NKG2D-Ls can be transferred from cancerous cells to NK cells after NKG2D/NKG2D-L ligation in a process known as trogocytosis (119, 120), although other authors associated this expression to interleukins such as IL-2 (121) or IL-12, IL-15 and IL-18 (122), depicting a non-fratricidal role but a recently activated mature phenotype.

In the context of CAR-NK cells, fratricide can also appear due to CAR-ligand/antigen recognition in CAR-NK cell surface as previously described in CAR-T cells (123–126). For instance, CD38 CAR-NK cells undergo fratricide since NK cells naturally express CD38 and its expression can be upregulated during *ex vivo* expansion in the presence of IL-2 or engineered feeder cells. Therefore, NK cells can destroy their siblings either after exposure to anti-CD38 antibody-based therapy (via ADCC) or by their recognition by an anti-CD38 CAR (127, 128). In a similar way, other antigens such as CD70 or CD33, which seem promising candidates to target hematological malignancies with CAR-NK cells, can be upregulated in NK cells during *ex vivo* expansion, depending on the employed stimulation protocol, entailing CD70 CAR and CD33 CAR-NK-mediated fratricide, respectively (129, 130).

Taken all together, it is strongly necessary to consider the most appropriate expansion method, the use of inhibitors or monoclonal antibodies during CAR-NK manufacturing, currently described for CAR-T cells (123), or even selecting NK donors with specific SNPs that avoid antibody or CAR recognition, as has been reported for CD33 (130). These strategies are emerging, together with CRISPR/Cas9-based gene editing that will be presented later in this review, to avoid fratricidal events that result in lower yields and diminished efficacy of CAR-NK cells *in vivo*.

### T cell allorejection

Donor NK cell recognition and rejection by the host immune system may potentially reduce allogenic CAR-NK cell persistence in the clinical setting. The primarily effectors responsible for these mechanisms are alloreactive T cells, which recognize non-self HLA molecules on allogeneic NK cells. Higher levels of exhausted T cells after lymphodepletion have been associated with a longer persistence of transferred haploidentical NK cells in leukemia patients (131). Lymphodepleting chemotherapy induces a transient reduction of the host immune system that improves adoptive cell engraftment. Alongside decreasing T and NK cells, lymphodepleting drugs also diminish cell populations that act as sinks for cytokines and/or have immunosuppressive properties, such as Tregs and myeloid-derived suppressor cells (MDSC), generating a more favorable microenvironment for adoptive cell expansion (132, 133). Miller et al. reported that high-dose of cyclophosphamide and fludarabine are required to achieve NK cell engraftment and expansion (134). Endogenous IL-15 increases after lymphodepleting treatment and has been associated with initial NK cell *in vivo* proliferation (134, 135). However, IL-15 together with IL-7 are essential for T cell homeostatic proliferation, which occurred after severe T cell depletion (133). Therefore, IL-15 may also contribute to CD8^+^ T cell allorejection (81). Exogenous cytokine support has been proposed to lengthen NK therapy persistence. Nevertheless, the IL-15 superagonist complex N-803 reduces clinical responses in AML patients treated with haploidentical ML-NK cells because of CD8^+^ T lymphocytes stimulation (NCT03050216 and NCT01898793) (136). Alternatively, autocrine secretion from bicistronic CAR constructs containing cytokines may provide a better approach. In that sense, IL-15-expressing CD19-CAR UCB NK cells have been detected for long-term post-infusion in non-Hodgkin’s lymphoma or CLL patients despite HLA-mismatching (22). Still, the optimal support to create the appropriate cytokine milieu that improves NK cell persistence minimizing T-cell-mediated allorejection has yet to be established.

Multiple NK cell infusions do not solve this issue because the persistence of NK cells from a second infusion is even shorter, suggesting a quicker allogenic response (137). Recently, two studies have reported that an immune-compatible clinical setting generated in the early post-HCT period may improve the persistence of allogeneic CIML-NK cells obtained from the same donor, due to the match of infused NK cells with graft-derived lymphocytes and absence of host alloreactive T cells (107, 109).

**Figure 2:**
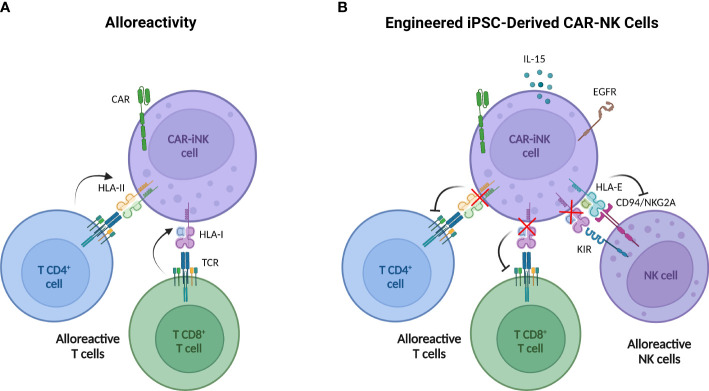
Multi-engineered iPSC-derived CAR NK cells designed to overcome T cell alloreactivity. **(A)** iPSC-derived CAR NK cells may be rejected by host alloreactive T cells due to the recognition of non-self HLA I by CD8+ T cells and HLA II by CD4+ T cells. **(B)** Engineered iPSC-derived CAR NK cells incorporate six modifications through three gene-editing steps. 1) The β-2-microglobulin (β2M) disruption to avoid HLA-I expression with the simultaneous insertion of a transgene encoding HLA-E protein (tethered with β2M and a peptide) impedes NK cell killing activity by “missing-self” recognition. 2) The CIITA knock-out to deplete the HLA-II expression concurrently with knock-in of the epidermal growth factor receptor (EGFR) safety switch and the interleukin 15 (IL-15). Safety switch strategy allows the elimination of iPSC-derived CAR NK by the administration of anti-EGFR antibodies and IL-15 secretion improves cell persistence. 3) CD19 CAR knock-in. Created with BioRender.com.

Currently, additional strategies are being developed to prevent host system rejection. Several approaches studied in other cell types are based on the expression of molecules that block the attack over the infused cell such as immune checkpoints (138), or on providing cells with receptors that favor the elimination of alloreactive T cells (139). In human PB-NK cells, Hoerster et al. have disrupted HLA class I expression by targeting the β-2-microglobulin gene (β2M) to circumvent CD8^+^ T cell alloreactivity. Simultaneously, a single-chain HLA-E molecule, which binds the inhibitory receptor NKG2A, has been overexpressed to avoid NK cell fratricide and host NK cell rejection by “missing-self” recognition (140). These modifications have been incorporated in CNTY-101 (**Figure 2**), a multi-engineered iPSC-derived CD19 CAR-NK product, in which class II major histocompatibility complex transactivator (CIITA) gene has also been disrupted to confer resistance to CD4^+^ T cell allogeneic response (141). Preclinical studies with both NK products reported that these genetic modifications do not compromise their antitumor potential.

## Surmounting replicative senescence: ‘Buying time’ for NK cells could enhance CAR-NK immunotherapy

NK cell short *in vivo* lifespan in the absence of cytokine support reduces off-tumor toxicities and malignancy risk but narrows the therapeutic window, abrogating long-lasting immunotherapy responses (142). *In vivo* persistence and proliferation of NK cells following adoptive transfer have been previously shown to correlate with clinical responses (143, 144). Therefore, low persistence *in vivo* could cause early relapses due to the disappearance of CAR-NK therapy. In addition, a short lifespan limits NK cell proliferation and expansion *ex vivo* during manufacturing, making it harder to achieve sufficient cell numbers for immunotherapy doses (55) and diminishing the time for NK cell optimization by genetic engineering. Consequently, an extension in the effector longevity may boost CAR-NK cell efficacy.

Unlike T effectors that can persist from months to a decade (145), human NK lifespan is not clearly defined, varies between subsets, and can be markedly manipulated *in vitro*. We and other groups reported that human primary NK or CAR-NK cells co-cultured with K562 aAPCs lines and cytokines typically promote log-phase NK cell expansion for up to 4 to 6 weeks without evidence of senescence (55, 142, 146). Primary NK cells activated by these feeder cells can eventually become unresponsive to stimulation and undergo senescence in a limit record of 15 weeks of continuous proliferation (147).

*In vivo*, mature NK cells require continuous cytokine support, without which they are detectable in the circulation for only 1-2 weeks (148). Expanded and activated human NK or CAR-NK only survived between 4-5 weeks in xenografted immunodeficient mice without any stimulation (142, 146) and up to 68 days when CAR-NK is engineered to express IL-15 (91).

In clinical use, Liu et al. found CD19-CAR UCB-NK cells by flow cytometry limited to the first three weeks even with lymphodepletion and IL-15-autocrine support. Nevertheless, DNA copies of the infused CAR were detected up to 12 months measured by real time-PCR in patients suffering CD19^+^ lymphoid tumors (22). Similarly, autologous NKAEs were detected by multiparametric flow cytometry around four weeks after infusion in MM clinical trial performed in a consolidation setting (46). In line with augmented lifespan bolstered by cytokines, CIML NK cells increased persistence up to 2-3 months analyzed by mass cytometry (107, 109), albeit CIML NK cells were administered in an “immune-permissive” microenvironment and combined with an IL-15 superagonist (107). Human CAR-NK effectors do not typically clonally expand *in vivo* like antigen-specific-T cells or virus-specific adaptive NK cells (149). They frequently peak in circulation between the first and second week post-transfer from where they progressively decline (22, 43, 107, 134). Together, these studies suggest that, although permissive to lifespan modification by exogenous cytokines or HLA matching, NK and CAR-NK are short-lived cells impacted by senescence that inexorably arises *ex vivo* as a consequence of expansion methods, and subsequently *in vivo*, where these effectors proliferate in a narrow window and do not persist long in patients.

Cellular senescence is a universal process considered a hallmark of aging and can be triggered in non-tumoral cells in response to different intrinsic and extrinsic stressors, as well as developmental signals. In particular, replicative senescence is related to loss of proliferative capacity and functional deficit characterized by the shortening of telomeres, the detection of genomic DNA double-strand breaks, the activation of repair machinery, and the arrest of the cell cycle to stop replication and prevent genomic instability (150). Eventually, senescent cells can surpass cell cycle checkpoints and enter in a crisis phase with augmented chromosomal and genomic instability, inducing apoptosis (151). T cell immunosenescence is a well-studied phenomenon observed during aging and prolonged *in vitro* cultures and differs from immune exhaustion by repeated stimulation. Terminal differentiated T effectors are characterized by CD28^-^ CD27^-^ KLRG1^+^ CD57^+^ CD45RA^+^ phenotype with shortened telomeres, active metabolic reprogramming, higher production of pro-inflammatory molecules (senescence-associated secretory phenotype), and less replicative ability (see review in Kasakovski et al.) (152). Although a phenotype of highly mature NK cell based on CD57 expression has been proposed, the NK cell immunosenescence field is still in its infancy with no phenotype and function clearly established (153, 154). Terminal NK cells are dysfunctional, identified by decreased NK effector functions, such as impaired ADCC, as well as reduced cytokine secretion like IFN-γ and expression of perforin and granzyme (155). Among the factors involved in NK cell longevity control, telomere length is critical because its shortening after multiple rounds of cell divisions (Hayflick limit) exposes the unstable chromosomal ends, initiates fusion-bridge-breakage cycles, and leads to genomic instability and replicative senescence. Human NK cells display telomere shortening and a reduction in telomerase activity with age (156). Cellular differentiation impacts telomere shortening, leading to the more mature CD56^dim^ NK cells having shorter relative telomere length than the immature CD56^bright^ subset (157).

Regarding adoptive NK therapy, telomere length depends on the NK source or the activation/expansion method selected. For example, telomere length in iPSC-derived NK cells is much longer compared to those expanded from PB (158). Yang et al. reported up-regulation of positive telomere length regulator genes such as ZNF257, LRRC34, NAF1, and human telomerase reverse transcriptase (hTERT) in NK cells expanded and activated with 721.221 feeder line with IL-2 and IL-15 (56). hTERT expression and activity are strictly regulated in somatic cells and can be reprogrammed by common gamma-chain cytokines, c-Myc (159) or fine-tuned by miRNAs (160, 161). Indeed, all IL-2, IL-15, and IL-21 have been shown to up-regulate telomerase activity in NK cells, thereby preventing telomere loss and allowing cells to extend replication. IL-2 increases telomerase activity in NK cells upregulating hTERT mRNA levels (162). MbIL-21 increased NK cell longevity by maintaining telomere length in K562 co-cultures (55, 163). In addition, IL-15 induces hTERT expression and cellular growth in NK culture *ex vivo* at lower doses than IL-2 (164). IL-21 is known to signal primarily through the STAT3 component of the JAK/STAT pathway, whereas IL-15 signals mainly through STAT5 (165).

Ectopic expression of hTERT by genetic engineering may be an effective strategy to improve CAR-NK cell persistence and thereby their therapeutic potential, paralleling seminal studies in CAR-T cells (166) where the maintenance of telomere length and replicative ability is associated with engraftment efficacy and antitumor efficiency (167). In a pioneer study from Campana´s lab, hTERT transfected NK and CD19 CAR-NK cells (expanded with K562-mb15-41BBL) restored replicative ability and could be cultured for almost one year with continued cytotoxicity against leukemic cell lines and exhibited normal karyotype (analyzed at day 186). However, transfected NK cells were not able to grow autonomously in nonobese diabetic severe‐combined‐immunodeficient γc−/− (NSG) mice and still eventually developed delayed senescence *in vitro* (147). More recently, Streltsova and coworkers corroborated that stable hTERT ectopic expression, even when gamma-retrovirus is used, increases the proliferation and lifespan of expanded and activated (K562-mbIL21+IL2) NK cells rather than complete immortalization (168). The safety of this strategy is a critical question because so far, the inability of ectopically expressed hTERT to cause oncogenic transformation of NK cells has not been firmly established. Further research is needed to attempt more refined approaches to overexpress hTERT in terms of expression control, for instance, inducible promoters or transient expression, as reported in CAR-T cells, which lead to improved proliferation and persistence in murine xenograft tumor models of human B-cell lymphomas (169). Another possible strategy is the implementation of safety switches in hTERT constructs to assure safety. Direct reprogramming of other components of telomere machinery by genetic engineering or by their stabilizers and/or manipulating telomere elongation factors could accelerate the translation of these strategies into clinical reality.

## Tumor microenvironment: The stumbling block that limits CAR-NK therapy effectiveness

**Figure 3:**
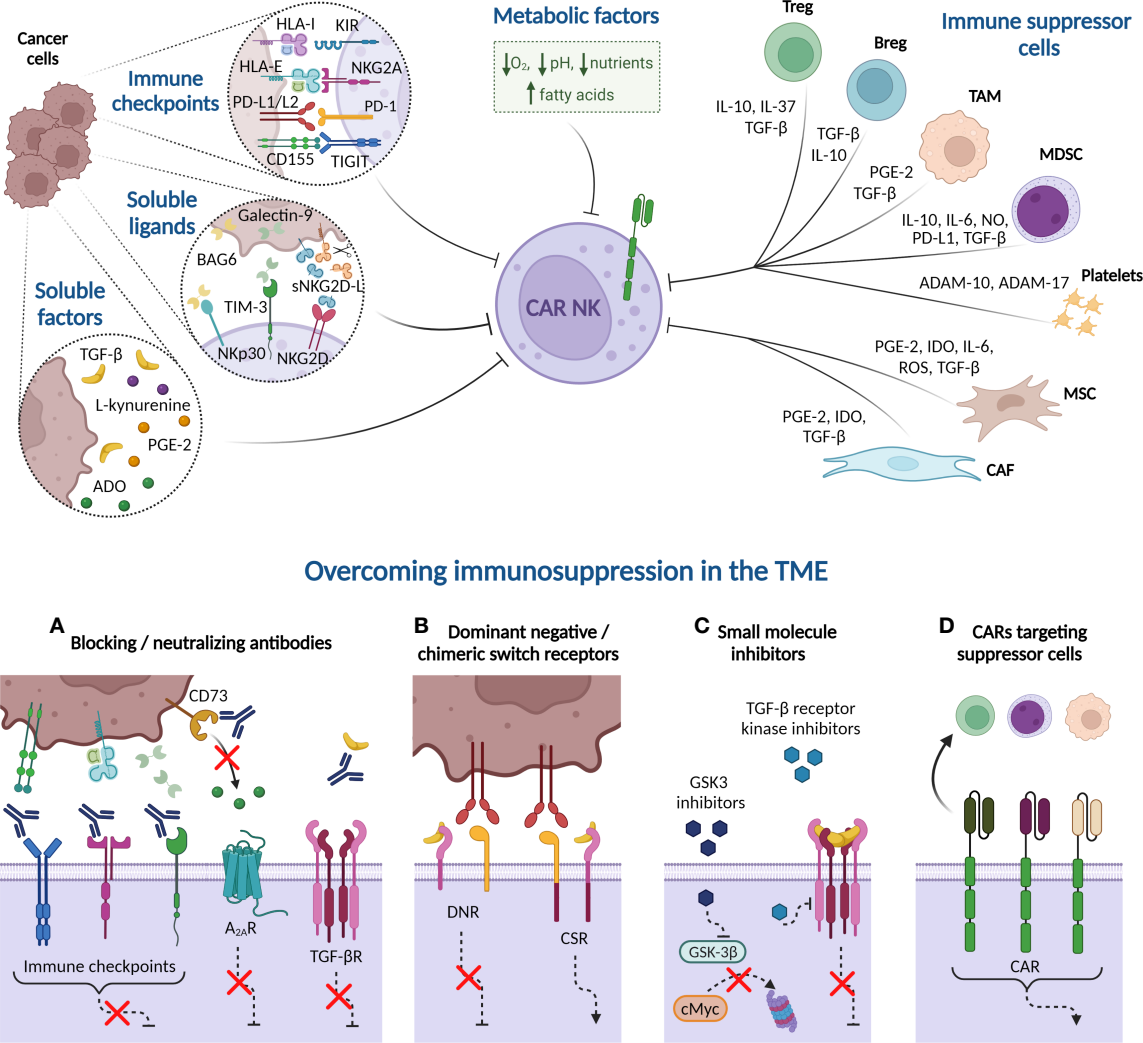
Immune suppressive tumor microenvironment (TME) factors potentially involved in CAR-NK cell dysfunction. Cancer cells express immune checkpoint ligands in their plasma membrane that mediate an inhibitory interaction with NK cells. Besides, tumor cells may suppress NK cell function by releasing soluble ligands to the milieu, such as BAG-6, galectin-9, and soluble NKG2D-L (sNKG2D-L), as well as other soluble factors, including cytokines, such as transforming growth factor-β (TGF-β), enzymes and metabolites. Many of these soluble factors are also produced by immune cells present in the TME, such as Tregs, Bregs, tumor-associated macrophages (TAM), and myeloid-derived suppressor cells (MDSC). Platelets, in turn, secrete the metalloproteinases ADAM-10 and ADAM-17 that prompt NKG2D-L shedding. Other non-immune cells, such as derived-mesenchymal stromal cells (MSC) and cancer-associated fibroblasts (CAF), also produce indoleamine 2, 3 dioxygenase (IDO) or reactive oxygen species (ROS) that reduce NK cell activity. Additionally, hypoxia, high concentrations of fatty acids, nutrient deprivation, and acidity, among other metabolic factors, contribute to generate a complex immunosuppressive TME that hampers the NK cell effectiveness against hematologic malignancies. Several strategies can overcome the immunosuppression mechanism from TME. **(A)** Blocking antibodies targeting immune checkpoints prevent the inhibition of NK cell cytotoxicity. Other receptors, such as adenosine A2A receptor (A_2A_R) also disable NK cell function when binds to extracellular adenosine (ADO). Blockade of CD73 ectoenzyme, which synthetizes ADO, reduces the levels of this metabolite in the TME, therefore increasing NK cell killing activity. Furthermore, anti-TGF-β neutralizing antibodies impede the NK cell suppressive effect unleashed by the interaction of this cytokine with its receptor (TGF-βR). **(B)** Dominant-negative receptor (DNR) expression hinders the inhibitory signaling triggered by PD-1 and TGF-βR in the presence of PD-L1/L2 or TGF-β, respectively. Chimeric switch receptors (CSR) constitutes another approach based on replacing these negative signals by activating ones, through intracellular domains exchange, reverting the outcomes in NK cell activity. **(C)** Small molecule inhibitors directed against GSK-3β impact on NK cell metabolism and improve their cytotoxic potency. Other inhibitors are engineered to inhibit the kinase activity of TGF-βR. **(D)** CAR constructs are designed against molecules expressed in immune suppressor cells to eliminate them from TME. HLA-I, HLA class I histocompatibility antigen; KIR, Killer-cell immunoglobulin-like receptor; HLA-E, HLA class I histocompatibility antigen, alpha chain E; PD-L1, Programmed Death ligand-1; PD-1, Programmed Death 1; TIGIT, T cell immunoglobulin and ITIM domain; BCL2-associated Athanogene 6 (BAG-6); sNKG2D-L, soluble natural killer group 2D ligands; TIM-3, T cell immunoglobulin and mucin-domain containing-3; PGE-2, prostaglandin E2; NO, nitric oxide; A Disintegrin And Metalloproteinase (ADAM). Created with BioRender.com.

As a result of overstimulation, tumor progression induces a reversible exhausted status in NK cells characterized by impaired effector functions and altered phenotype, similar to previously described in T cells. The expression of tumor-associated immune checkpoints reduces NK cell killing activity *via* direct cell-cell interactions. NK cell *ex vivo* expansion also fosters the expression of some immune checkpoint receptors, potentially decreasing CAR-NK therapy efficacy (**Figure 3**). On top of that, patients’ NK cells and infused CAR-NK cells encounter a hostile microenvironment in the tumor niche, generated by immunosuppressive cells and soluble factors, which leads to NK cell suppression. Consequently, many efforts are underway to identify and neutralize the negative TME factors that may limit CAR therapy effectiveness.

### Exhaustion-associated immune checkpoints

Classical inhibitory receptors of NK cells, such as KIRs and NKG2A act as immune checkpoints (see major immune checkpoints in **Figure 1**). In many malignancies, tumor cells downregulate the expression of classical HLA-I molecules, preventing Ag-dependent recognition by T cells but allowing NK cell “missing-self” activation (170, 171). By contrast, in some hematological tumors such as MM, classical HLA-I expression is elevated in advanced stages (172), inducing KIR-mediated NK cell inhibition. In preclinical studies, the use of anti-KIR antibodies recovers NK cell killing activity (173, 174), but the administration of pan-KIR2D antibodies, such as IPH2101 or lirilumab (IPH2102), has not shown single-agent activity in clinical trials of MM (175) or AML (176). Unexpectedly, IPH2101 infusion in smoldering MM patients induces NK cell anergy due to the removal of KIR2D surface molecules through trogocytosis (175). In addition, a phase II trial reports that lirilumab administration as maintenance therapy for elderly AML patients fails to improve leukemia-free survival (LFS) (NCT01687387) (176). For that reason, combined therapies with drugs, such as IMiDs, or other immune checkpoints blocking antibodies are being examined (177). IPH2101/lenalidomide dual therapy has shown a synergistic effect against MM in preclinical studies and preliminary evidence of efficacy in phase I clinical trial (NCT01217203) (174, 178). Similarly, the same synergic strategy is being evaluated in studies of anti-KIR antibodies combined with anti-PD-1 and anti-CTLA-4 blocking antibodies against solid tumors (NCT01750580, NCT01714739, NCT03203876). Many studies have focused on the NKG2A receptor that triggers inhibitory signaling upon binding HLA-E, a non-classical HLA-I molecule overexpressed in several tumors (179). Approximately half of peripheral blood NK cells from healthy donors express NKG2A (180, 181) and their levels increase after *ex vivo* stimulation with cytokines (182). NKG2A trapping in the endoplasmic reticulum/Golgi by smartly designed protein expression blockers (PEBLs) (183) or the treatment with anti-NKG2A blocking antibodies, broadens the oncolytic activity of NK cells against hematological tumors such as AML, CLL or lymphomas (184). Monalizumab (IPH2201), a humanized monoclonal anti-NKG2A blocking antibody, has also shown promising preclinical results against hematologic and solid tumors (185, 186), which have prompted to evaluate its efficacy in clinical trials either as monotherapy or combined with other treatments (187). Other strategies to inhibit the NKG2A/HLA-E axis are based on NKG2A downregulation in NK cells by dasatinib (188), or the reduction of HLA-E levels in tumor cells, by drugs such as bortezomib, dinaciclib or selinexor, tested *in vitro* on MM, AML, and lymphoma, respectively (189–191). Mechanistically, dasatinib treatment inhibits p38 mitogen-activated protein kinase (MAPK), diminishing the import of NKG2A transcription factor GATA-3 to the cell nuclei (188). Meanwhile, HLA-E downregulation by bortezomib is induced through endoplasmatic reticulum-stress unleashed by proteasome inhibition (189). Constant *de novo* protein synthesis is essential for maintaining HLA-E surface expression levels. Consequently, selinexor induced degradation of nuclear export protein exportin-1 (XPO1), which regulates the transport of ribosomal subunits from nucleus to cytoplasm, decreases the number of HLA-E molecules in plasma membrane, mainly because of the reduction of HLA-E binding substrates (191). Although the action mechanism of dinaciclib has not yet been elucidated, the administration of this cyclin-dependent kinase (CDK) inhibitor prior to NK cell infusion further boosts their killing activity in an AML mouse model (190). Due to their antitumor function, bortezomib, dinaciclib, and selinexor are being used in different hematological pathologies, but besides, their administration as a pretreatment before adoptive cell therapy could enhance CAR-NK cell efficacy.

Combining CAR-T cell therapy with Programmed Death (PD)-1/PD-L1 axis blockade has improved clinical responses in hematological tumors (192). Although NK cell inhibition by PD-1/PD-L1 checkpoint has been reported (193, 194), *ex vivo* expanded NK cells exhibit very low PD-1 expression levels, hence the combination with pembrolizumab does not improve *in vitro* cytotoxicity (195–197). However, some studies suggest that this immune checkpoint may become important post-NK therapy infusion because expanded NK cells increase PD-1 expression in the presence of tumor cells, and IFN-γ produced by NK cells augments PD-L1 expression in a lung cancer mouse model (198). In addition, trogocytosis has been described recently as a new mechanism whereby NK cells obtain PD-1 from tumor cells (199). A phase II clinical trial (NCT04847466) combining PD-L1 CAR-NK cells with pembrolizumab and N-803 is currently being studied in gastric and head and neck cancer. Similar to NKG2A, NK cell *ex vivo* expansion upregulates the expression of other exhaustion receptors, such as T cell Ig and mucin-containing domain-3 (TIM-3) (200) and T cell immunoreceptor with Ig and ITIM domains (TIGIT) (197). There is controversy regarding the role of TIM-3 in NK cell activity. Although most studies define TIM-3 as an NK cell checkpoint inhibitor, some papers show that TIM-3 interaction with its ligands (including Galectin-9) unleashes IFN-γ production or associate TIM-3 expression with a functionally licensed NK cell phenotype with higher cytotoxic activity (201, 202). A functional threshold that controls the activatory or inhibitory NK cell function of this receptor has been proposed to explain this discrepancy. Meanwhile, TIGIT recognizes poliovirus receptor (PVR or CD155), Nectin-2 (CD112), or Nectin-3 overexpressed in hematologic cancers (203, 204). The blockade of TIM-3 or TIGIT in preclinical studies improves NK cell cytotoxic potency against solid and hematologic malignancies (205–209) and currently neutralizing antibodies are being tested in several clinical trials (e.g. NCT04623216, NCT03489343, NCT04150965, NCT04354246, and NCT05289492). Nevertheless, the results obtained from these studies should be analyzed considering that TIM-3 and TIGIT are also expressed in T cells, making difficult to determine the precise involvement of the NK population in the patient response. Nectin-2 is also recognized by PVRIG (CD112R) a novel T cell inhibitory receptor that does not bind CD155 (210). Studies in AML suggest that PVRIG impacts NK cell cytotoxic activity against CD112^high^CD155^low^ tumors, whereas, in those with overexpression of both ligands, TIGIT inhibitory signaling is predominant (211). Unlike in T cells, expression levels and inhibition relevance of other receptors such as LAG-3 or CTLA-4 remain unclear in NK cells (212, 213).

Additional molecules are still being included within the immune checkpoint category. For instance, signal regulatory protein α (SIRPα) is a myeloid-lineage receptor recently described in T and NK cells, whose expression increases after IL-2 stimulation (214, 215). SIRPα deficiency or blockade enhances NK cell cytotoxicity against CD47-expressing tumor cells (214), which include hematological malignancies, such as diffuse large B-cell lymphoma (216). Recently, it has been described that many tumor proteins are hypersialylated, a modification that confers cancer cell resistance to the cytotoxic activity of different immune populations through their recognition by inhibitory sialic acid-binding immunoglobulin-like lectin (Siglec) receptors (217). Deletion of Siglec-7 or blockade of Siglec-9 restores NK cell cytotoxic activity against MM (218) or CML cells, respectively (219).

Taken together, not all the immune checkpoints are induced at the same levels in *ex vivo* expanded NK cells neither are they similarly relevant in the modulation of NK cell antitumor activity. The balance of activating and inhibitory signals regulates NK cell function; therefore, more efforts are needed to evaluate the impact of each immune checkpoint expression in the presence of CAR stimulation to direct the strategies to improve CAR-NK therapy.

### Suppression in the TME

Soluble factors from TME contribute to heightening NK cell inhibition. Tumor cells release soluble ligands that can bind activating and inhibitory receptors expressed in NK cells to promote their dysfunction. Soluble NKG2D-L (sNKG2D-L) generated by proteolytic shedding decrease the expression of NKG2D, reducing NK cell antitumor potency (220, 221). Interestingly, sNKG2D-L do not impair the effectiveness of NKG2D-CAR NK92MI nor NKG2D-CAR T cells against MM and leukemia cells, respectively (222, 223). Specific antibodies directed against the MICA α3domain inhibit the shedding of this NKG2D-L, allowing NK cell-mediated cytotoxicity and thus avoiding tumor evasion (224). CAR constructs designed to recognize the same MICA/B domain have shown efficacy against leukemia in iPSC-derived NK cells and are currently being studied in a dual-CAR in combination with BCMA specificity for MM (225, 226). Likewise, the presence of BCL2-associated Athanogene 6 (BAG-6), one of the NKp30 ligands, in the tumor cell membrane or exosomes stimulates NK cell antitumor activity whereas its soluble form hampers NK cell function (227, 228). Other soluble ligands such as galectin-9 have been found at high levels in the blood plasma of AML patients (208). Galectin-9 effect has not been studied in CAR-NK cells yet, but its blockade reestablishes CAR-T cell antitumor responses (229).

Additionally, well-known soluble factors present in the TME of most cancers, such as certain interleukins, enzymes, and metabolites impact NK cell effectiveness. Most of them are released not only by tumor cells but also by immune suppressor cells that coexist in the tumor niche. That is the case of IL-37, produced by Tregs, which exerts an inhibitory action over canonical NK cells while adaptive NK cells are highly resistant (230). High concentrations of other suppressive cytokines, such as IL-6, IL-10, and TGF-β have been widely reported in hematologic tumors (231–234). Concerning NK cells, TGF-β favors tumor development by decreasing activating receptor expression, cytokine production, and metabolism (235–237). Alongside the anti-TGF-β neutralizing antibodies or engineered NK cells to knock-down TGF-β receptor expression, other strategies such as small molecule receptor kinase inhibitors can be implemented for CAR-NK cell therapy in hematological cancers (238, 239). For instance, *ex vivo* expanded NK cells restored their in vitro anti-AML activity by the addition of TGF-β receptor kinase inhibitor LY2157299 (235). TGF-β is affected by prostaglandin E2 (PGE-2), which is secreted by stromal cells derived from lymph nodes (LN) or bone marrow (BM) as well as by some tumor cells, such as leukemic blasts, sustaining MM and leukemic cell proliferation (240–242). The binding of PGE-2 to its receptor in monocytes induces an “MDSC-like” phenotype that enhances TGF-β production. In this manner, PGE-2 can interfere with NK cell function in two different ways, directly, joining to its receptors in NK cells, and indirectly, enhancing TGF-β concentrations in the TME (243, 244). Furthermore, TGF-β raises the expression of CD39 and CD73 in cancerous and other cell types (245, 246). These ectoenzymes are involved in adenosine (ADO) production from ATP/AMP, promoting tumor proliferation. ADO inhibits NK cell activity and expansion and reduces its metabolism (247–249). In fact, combination therapy of NKG2D-CAR-NK cells with anti-CD73 blocking antibody increase NK-92 cell killing potency against CD73^+^ cancer cells, reducing ADO levels in the TME (250). By the same token, the activity of indoleamine 2,3-dioxygenase (IDO) downregulates NKp46 and NKG2D and restricts NK cell cytotoxicity (251). IDO is an enzyme that degrades tryptophan to L-kynurenine and its inhibition reestablishes NK cell activity (252). IDO expression has been associated with poor prognosis in AML, diffuse large B-cell lymphoma as well as solid tumors (253–256). Apart from the effect of the tryptophan-derived suppressive catabolite L-kynurenine (251), IDO contributes to decreasing NK cell proliferation by depriving tryptophan in the tumor milieu. Similarly, high concentrations of arginase, released by immune suppressor cells, decrease arginine levels, essential for T and NK functions, which can be restored by arginase inhibitors (257, 258).

In addition to the aforementioned strategies to prevent NK cell inhibitory interactions with cancer cells by immune checkpoints or with immunosuppressive soluble factors, dominant-negative receptors have also been designed (259–261). The expression of a dominant-negative TGF-βRII (DNRII) in UCB-NK cells allows the maintenance of the NKG2D and DNAM-1 expression levels and glioblastoma cell-specific lysis in the presence of TGF-β (260). Other approaches are based on converting inhibitory into activating signals by chimeric switch receptors (CSR), also known as chimeric costimulatory converting receptor (CCCR). Tested so far in the context of solid tumors, CSR directed to PD-1, B7-H3 or TGF-β increases the anti-tumorigenic capacity of NK92 cells (262–264). Moreover, TGF-βRII-NKG2D receptor expression promotes NK92 chemotaxis to the TGF-β-expressing tumor cells and inhibits the differentiation of CD4^+^ T cells to Tregs (264).

Immune suppressor cells present in the TME promote tumor proliferation while reducing NK cell function through direct cell-cell contact or by releasing soluble factors. The detrimental effect of Tregs on NK cells through these mechanisms and the previously mentioned competition for IL-2 are widely known (67, 265, 266). Other immune cells that contribute to NK cell suppression such as Bregs, MDSCs, and tumor-associated macrophages (TAM), mainly M2 phenotype, are highly represented in the TME (267–271). An additional impediment to NK cell function is mediated by platelets which stunt NK cell recognition (272, 273). In addition, platelets favor NKG2D-L shedding due to A Disintegrin And Metalloproteinase (ADAM)-10 and ADAM-17 release. Platelets’ blockade enables NK cell access to the tumor and recovers activating receptor expression on the NK cell membrane (274–279). Furthermore, non-immune cells present in the TME, such as derived-mesenchymal stromal cells (MSC) and cancer-associated fibroblasts (CAF), strengthen NK dysfunction because they impair degranulation, reduce perforin and cytokine secretion and hinder antitumor efficacy (280, 281).

Such evidence have led to developing CAR therapies to eliminate these suppressor cells. In hematological cancers, CAR-NK cell products have been designed to target Tregs (directed against CD25 or CD38) (282, 283) or MSCs (against CD38) (284) to restore tumor-infiltrating NK cell activity. Interestingly, other CAR-NK cell strategies take advantage of the ligand shared expression between cancer cells and MDSCs, such as NKG2D-CAR-NK cells and PD-L1-CAR-NK cells that can kill both cell types (285, 286). Similarly, dual FAP/SLAMF7-CAR-T therapies are novel approaches for targeting CAFs, surmounting the suppressor function of these cells over CAR effectors, and eliminating MM cells at the same time (287). Meanwhile, TAM-specific CAR-T cells, targeting folate receptor β (FRβ), reduce the number of TAMs in the TME while decreasing tumor cells’ proliferation rate. Therefore, this adoptive treatment has been suggested to be administered before tumor-directed CAR-T cells (288). These two last approaches in CAR-T cells could also be implemented to create a more benign milieu for CAR-NK cell therapy.

Hypoxia and metabolic factors, such as nutrient deprivation, and acidity also generate an unfavorable microenvironment that compromises NK cell antitumor activity. Hypoxia is a well-described protumor factor in solid cancers and a feature of LN and BM microenvironment (289). Low O_2_ concentrations hamper the upregulation of activating receptors in NK cells (290, 291) while increasing MICA shedding (292, 293) and granzyme B degradation by autophagy in malignant cells (294). Additionally, hypoxia induces CD73 expression through hypoxia-inducible factor-1α (HIF-1α) (295), contributing to achieving high ADO levels in the TME. Upon stimulation, NK cells suffer a metabolic reprogramming regulated by mammalian target of rapamycin complex 1 (mTORC1), elevating oxidative phosphorylation (OXPHOS) and glycolysis and increasing the expression of glucose and amino acid transporters (296–299). Tumor cells require a greater amount of nutrients to proliferate, thus they compete for these resources with surrounding immune cells. Depriving NK cells of glucose or certain amino acids hinders the NK cell metabolism by inhibiting mTORC1 and cMyc pathways (296, 300). cMyc is key for NK cell metabolic responses because is involved in regulating the expression of glucose transporters and glycolytic enzymes (300). The levels of cMyc are initially controlled by mTOR and sustained through amino acid availability, to counteract its rapid rates of proteolysis by glycogen synthase kinase (GSK)-3β (301). Furthermore, GSK-3β inhibition increase *ex vivo* expanded NK cell antitumor activity in an AML mouse model (302), adding another pathway to aim CAR-NK cell improvement. Because cancer cells obtain energy mainly through high rates of glycolysis converting glucose into lactic acid, the latter is accumulated in the TME contributing to its acidification. Lactic acid diminishes NK cell cytokine production and induces apoptosis, in addition to increasing the number of MDSC (303, 304). In a lymphoma mouse model, oral delivery of bicarbonate restores NK cell IFN-γ production and tumor infiltration, although other tumor-dependent mechanisms preclude NK cell cytotoxicity (305). Additionally, tumor cells increase lipid metabolism, and fatty acid exposure hampers cytokine production and cytotoxic activity of NK cells (306). High levels of intracellular lipids trigger PPAR-γ/PPAR-δ signaling that aids NK cells in adapting to the altered milieu but inhibits the sterol regulatory element binding protein (SREBP). SREBP has been reported to be essential to regulate glucose metabolism (307, 308).

Considering the above, soluble factors, suppressor cells, as well as metabolic factors can induce NK cell dysfunction and should be taken into account when designing a new CAR-NK therapy. Overcoming the TME could guarantee longer and deeper CAR-NK responses in cancer patients. Studies with CAR-T cells and non-engineered NK cells have provided relevant information to take the first step, but specific assays with CAR-NK cells in hematologic tumors must be performed to maximize the results.

## Restoring migration and homing into tumor bed: A matter of chemotaxis

One of the challenges of CAR immunotherapy lies in limited trafficking and homing ability to reach the BM and LNs which is ultimately related to the exerted efficacy of adoptively transferred NK cells. Clinical studies suggest that improved trafficking of adoptively infused NK cells into BM niches is associated with better control of the disease in AML patients (143, 144), which could apply to other malignancies residing in the BM. A variety of strategies to maintain and/or enforce the expression of chemokine or adhesion receptors in CAR-NK cells are now being explored in preclinical models to improve their localization into the BM and LN.

CXCR4, CXCR3, CCR3, CCR5, and CX3CR1 are the main chemokine receptors expressed by NK populations that contribute to NK distribution in response to chemokines present in the TME (309). CXCL12 chemokine, the CXCR4 ligand, is highly expressed in MM by endothelial and BM stromal cells (310, 311), as well as in leukemia and lymphoma (312). Similarly, the BM niche of ALL (313), AML (314), lymphoma (315, 316), and MM patients is frequently characterized by upregulation of CXCL9 and/or CXCL10 (317). Of note, CXCL9 and CXCL10 (CXCR3 ligands) are considered immunosuppressive chemokines involve in MM resistance mechanisms (318). Additionally, macrophage migration inhibitory factor (MIF) can also bind to CXCR4 and CXCR7 and it was also found in high levels in MM BM (319). CCL19 and CCL21, chemokine ligands of CCR7, participate in the entry of CLL cells (320) into LNs, where they are found (321, 322).

Taking into account the high levels of chemokines found in the TME of hematologic malignancies, modifying the expression of chemokine receptors in adoptively transferred NK cells seems to be a suitable strategy. CXCR4 expression levels are higher in UCB-derived NK cells than those in PB-NK cells, suggesting a better BM homing ability (323). CXCR4 is generally downregulated during *ex vivo* activation of NK cells (317, 324–327), resulting in decreased homing to BM. Thus, some strategies are studying how to improve NK trafficking to this tumor niche. Human CD19-CAR-NK cells modified to overexpress CXCR4 through bicistronic lentiviral transduction augmented more than two-fold the migration to CD19^+^ tumor cells compared to huCAR19 NK cells (328). Ectopic expression of gain-of-function (GOF) mutation in CXCR4 (CXCR4^R334X^) *via* mRNA transfection on *ex vivo* expanded NK cells resulted in improved chemotaxis toward CXCL12 (also known as SDF-1α) and superior *in vivo* migration to BM (324). Similarly, the overexpression of the CXCR4^R334X^ receptor *via* electroporation on *ex vivo* expanded BCMA-CAR-NK cells was effective in enhancing the *in vivo* migration toward the BM and also eliminating myeloma cells in mice with prolonged survival as compared with only anti-BCMA-CAR-NK cells (327). Conversely, CXCR3 can be responsible for NK mobilization outside the BM (317, 329). This negative role of CXCR3 can be reversed by genetic deletion of Cxcr3 gene or by using an anti-CXCR3 blocking mAb to increase BM NK cell infiltration (317).

CCR7 is known to promote NK cell migration to LNs in response to chemokine ligands CCL19 and CCL21 (330). Its downregulation following *ex vivo* expansion has also been reported (325) but can be restored upon treatment with IL-18 (331). Besides, the transfer of CCR7 receptor from antigen-presenting cell (APC) to NK cell through trogocytosis can occur when co-cultured (332). In this line, LN homing of adoptively transferred NK cells can be enhanced by the *ex vivo* acquisition of CCR7 *via* trogocytosis from K562 feeder cells in athymic nude mice (333). Similarly, CCR7 mRNA-electroporation has been used to promote migration toward CCL19 and CCL21 chemokines (330, 334). CCR7 transfected anti-CD19 CAR-NK cells enhance their capacity to kill CD19^+^ tumors up to 5-fold increase and their migratory capability in response to CCL19 and CCL21 chemokines up to 6-fold increase (334). Schomer et al. have recently corroborated in CCR7 engineered CD19 CAR t-haNK cells an improved migration toward LN chemokine CCL19 compared to only CD19-CAR t-haNK cells in an *in vivo* xenografted NSG lymphoma model (335).

A novel strategy knocking-down CCR5 in *ex vivo* expanded NK cells reduces sequestration by the liver following i.v. infusion of adoptive immunotherapy which, in turn, favors their presence in the circulation (325). Given that CCR5 expression augments upon *ex vivo* expansion of adoptively transferred NK cells, this strategy bolsters the capacity to redirect NK trafficking *in vivo*. Levy et al. have opened the way to explore whether combining the overexpression of CXCR4 and CCR5 deletion would boost NK cell immunotherapy migration to the tumors residing in the BM (325), or even into the LN.

Apart from strategies modulating the expression of chemokine receptors, BM homing can be enhanced by promoting the interaction of NK cells with adhesion molecules like E-selectin. For instance, the treatment of NK-92MI cells with human fucosyltransferase 6 (FUT6) and GDP-fucose creates cell-surface E-selectin ligand sialyl Lewis X (sLeX) to improve migration to BM and increase the killing of B-lymphoma cells (336). Besides, while augmenting NK cell expansion in culture, nicotinamide (NAM) leads to CD62L (L-selectin) upregulation (337) resulting in improved *in vivo* migration of adoptively transferred NK cells to multiple organs including the BM (338, 339).

Most migration studies are being carried out for solid cancers as NK penetration into the tumor site is more critical than in liquid malignancies. Nevertheless, many of the chemokine ligand-receptor axes that are studied in solid tumors can be implemented in hematologic malignancies. An NK-cell-recruiting protein-conjugated antibody (NRP-body) that includes a cleavable CXCL16 molecule, the CXCR6 ligand, has been used to increase the trafficking and infiltration of expanded NK cells into pancreatic tumors (340). Additionally, CXCR1 and CXCR2 chemokine receptors display a high affinity for IL-8 (CXCL8), secreted by tumor cells in different cancer types. Although NK cells lose expression of these receptors upon activation and expansion (341, 342), their upregulation promotes enhanced attraction to tumor sites. Ng et al. show that CAR-NK cells expressing an NKG2D-CAR and CXCR1 chemokine receptor augmented their migration ability toward IL-8-secreting ovarian tumors and enhanced *in vivo* tumor control (341). Similarly, CXCR2-transduced NK cells have an increased ability to migrate toward renal cell carcinoma tumors in a ligand-specific manner, resulting in increased killing of target cells (342). As long as CXCL16 is found in the BM at high levels (343) and CXCL8, the CXCR1, and CXCR2 ligand is significantly elevated in MM (344, 345), CLL (346), AML, and MDS patients (347), the targeting of CXCL16-CXCR6 and IL8-CXCR2/CXCR1 pathways should be studied in depth for hematologic malignancies.

## Genome editing: Designing the CAR-NK 2.0

Genome editing promotes desired modifications in the specific locus of the genome by the use of engineered nucleases that activate endogenous DNA repair mechanisms. Although different engineered nucleases have been developed, the CRISPR/Cas9 system has completely revolutionized the field (348, 349).

CRISPR/Cas9 type II system, the most widely applied, is composed by an RNA-guided endonuclease (Cas9 nuclease) and a guide RNA (gRNA) that will direct the Cas9 nuclease to the desired locus. Cas9 and gRNA complex can specifically bind to the target sequence when protospacer adjacent motif (PAM) sequence is present (350) and generate a double-strand break (DSB) in the aimed region. Then, this DSB can be repaired by the endogenous DNA repair mechanisms of the cells, and consequently lead to the interruption of the gene, when Non-Homologous End Joining (NHEJ) or microhomology-mediated end joining (MMEJ) mechanisms occur, or the correction of a specific sequence when a donor DNA molecule with homology regions is present by homology-directed repair (HDR) (351). Importantly, NHEJ is the preferred mechanism to repair DSBs in mammalian cells, complicating the application of gene editing mediated by HDR in human cells (352).

Moreover, gene modification of NK cells using gene therapy delivery vectors such as plasmids and lentiviral vectors is particularly arduous, due to innate immune mechanisms that confer them resistance to genetic modifications (353, 354). This indicates that knock-in approaches, in which the delivery of a donor template requires the use of different types of vectors or plasmids will be challenging in these cells. In contrast, knock-out strategies using pre-transcribed gRNA and Cas9 protein in ribonucleoprotein complexes can potentially avoid problems associated with NK resistance to viral transduction and double-strand DNA (355–357).

**Figure 4:**
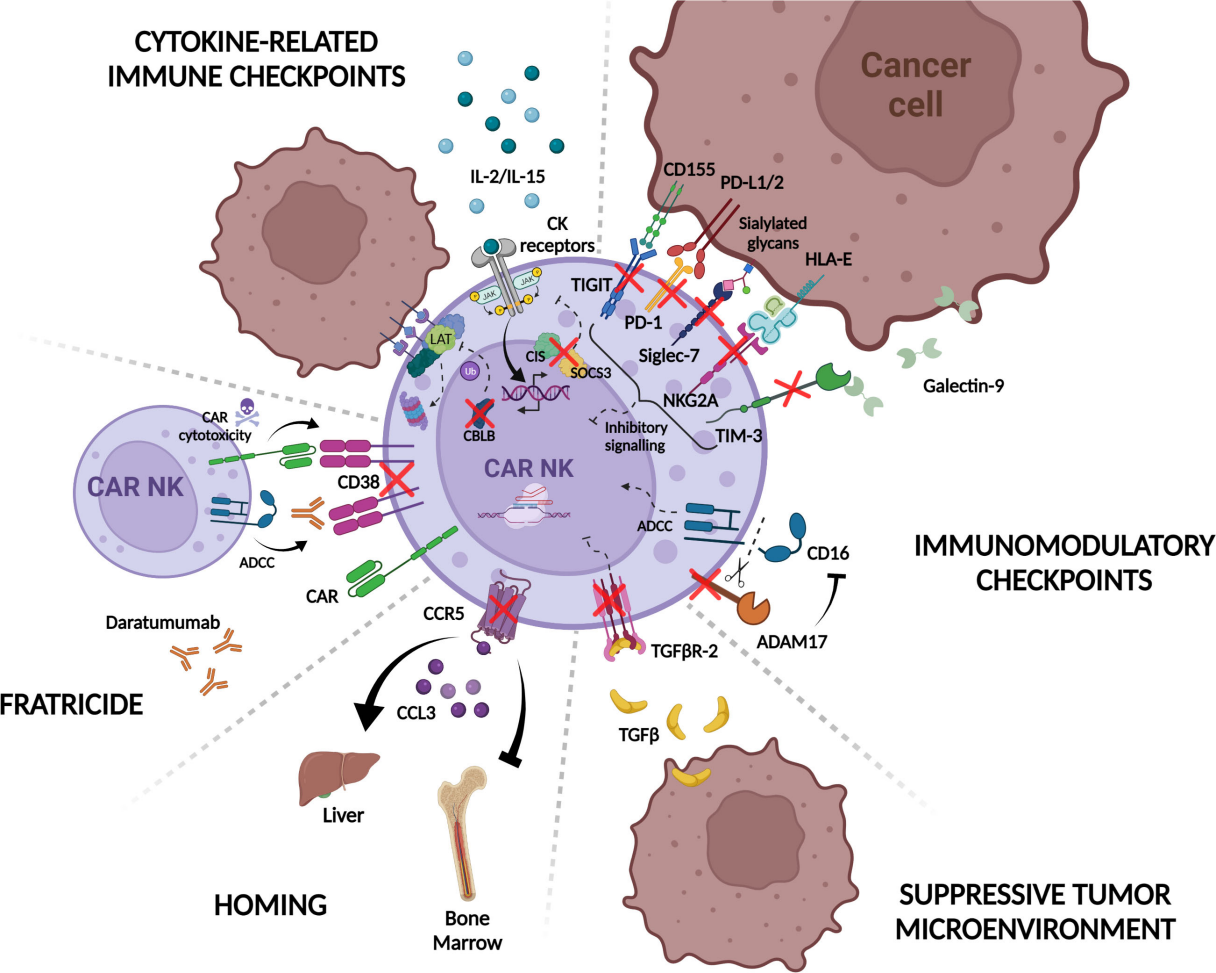
CRISPR/Cas9 knock-out strategies to improve CAR-NK immunotherapy. CAR-NK function and cytotoxicity are modulated by intrinsic mechanisms in NK cells. For example, engagement of tumor ligands with NK immunomodulatory checkpoint receptors such as TIGIT, PD-1, NKG2A, TIM-3, and Siglec-7, inhibit CAR-NK cell response to target cells. ADAM17 also restrains NK cell ADCC response by shedding CD16 receptor from the NK cell surface. Additionally, in response to cytokine signaling, the expression of internal checkpoints including CLBL, SOCS3, and CIS regulate NK activation and immune synapsis formation with tumor cells. The suppressive tumor microenvironment contributes as well to CAR-NK inhibition through the release of suppressive factors like TGF-β. In this context, CAR-NK potency is attenuated and less effective against tumor cells. CAR-NK cell homing is also regulated by chemokine receptors such as CCR5 that mediates homing to the liver in response to CCL3 reducing CAR-NK efficacy against bone marrow-resident tumors. Another problem that CAR-NK manufacturing can encounter is fratricide either by expression of the CAR-targeted molecule on the surface of the effector cell or the use of monoclonal antibodies that bind to NK cells and induce “self-killing” through ADCC. Ablation of different NK cell proteins implicated in these pathways by the use of the CRISPR/Cas9 system (red crosses) would overcome the aforementioned limitations and result in more potent, persistent, and tumor-directed CAR-NK effectors for their use in adoptive immunotherapy. PD-1, Programmed Death 1; TIGIT, T cell immunoglobulin and ITIM domain; TIM-3, T cell immunoglobulin and mucin-domain containing-3; NKG2A, natural killer group 2A; PD-L1/2, Programmed Death ligand-1/2; HLA-E, HLA class I histocompatibility antigen, alpha chain E; ADAM-17, A disintegrin and metalloprotease 17; TGF- β, Transforming growth factor beta; TGFβR-2, Transforming growth factor beta receptor type 2; CCL3 Chemokine (C-C motif) ligand 3; CCR5, C-C chemokine receptor type 5; CAR, chimeric antigen receptor; ADCC, Antibody-dependent cellular cytotoxicity; CK, cytokines; CIS, cytokine-inducible SH2-containing protein; SOCS3, suppressors of cytokine signaling; LAT, linker for activation of T cell; CBLB, Casitas B-lineage lymphoma protooncogene B Created with BioRender.com.

The application of gene editing constitutes nowadays the great hope to improve NK efficiency and persistence against different types of tumors. To this aim different studies have been focused on identifying negative regulators that could be targeted to modulate immune function and enhance NK and CAR-NK potency, either by increasing cytotoxicity, improving metabolism and *in vivo* persistence, or by overcoming mechanisms of functional exhaustion triggered by inhibitory immune checkpoints and TME (**Figure 4**).

One of the main strategies to improve CAR-NK persistence is focused on targeting inhibitory immune checkpoints such as PD-1/PD-L1 axis and in fact, PD-1 knockout (KO) in NK cells increased their antitumor activity in a xenograft model of ovarian cancer (358). The same authors tested the efficacy of PD-1 KO NK cells against CML and AML cell lines *in vitro* (358). Despite controversy about the low expression of PD-1 in *ex vivo* expanded NK cells, the enhanced response with PD-1 blocking antibodies in MM (359), suggests that disruption of this receptor could be a promising strategy to potentiate CAR-NK efficacy against hematological malignancies.

TIM-3 is another checkpoint receptor expressed by NK cells as a marker of dysfunction when TIM-3 positive NK cells encounter tumors expressing TIM3 ligands such as glioblastoma (360, 361) or AML blasts (208). Consequently, TIM-3 KO NK cells mediated improved cytotoxicity *in vitro* (362). A similar CRISPR/Cas9-based strategy has been applied for Siglec-7 receptor, which triggers NK inhibition when binds to certain sialylated glycans expressed on tumor cells, resulting in enhanced NK antitumor efficacy against Siglec-7 ligand-expressing MM cell lines (218).

Several groups have shown that TIGIT can inhibit immune response mediated by T and NK cells, leading to tumor escape. Thus, blocking TIGIT with mAbs is translated to an increase in NK persistence and antitumor capacity (363). More recently, TIGIT KO by gene editing has also been performed (355, 356). Although functional studies in TIGIT KO NK cells have not been described in this study one can envision that similar results could be obtained.

Targeting inhibitory signals in NK cells, by the use of KIR (NCT01714739 and NCT01750580) or NKG2A inhibitors (NCT02331875) is another interesting strategy to increase NK efficiency against hematological and solid tumors. Moving to CRISPR/Cas9 mediated editing approaches, targeting HLA-E/NKG2A axis by knocking out NKG2A-encoding killer cell lectin like receptor C1 (*KLRC1*) in CIML NK or NK cells has been described to induce a heightened response measured by IFN-γ production or increase cytotoxicity against AML or primary MM cells, respectively (364, 365). However, in the context of CAR-NK cells, it is still not clear whether ablation of this inhibitory receptor would provide an advantageous feature to the effector cells, considering the results indicating that NKG2A-KO CAR-NK92 cells and iPSC-derived CAR-NK cells do not show enhanced *in vitro* cytotoxicity against both solid and liquid tumor cell lines (263, 366). These results suggest that NKG2A not only constitutes an inhibitory receptor but also impacts during NK “education” process (366).

Alternatively, strategies focused on blocking regulators of NK activators such as ADAM-17 that cleaves CD16a, the NK cell-mediated ADCC activating receptor, have been tested to enhance antitumor response against a Burkitt’s lymphoma cell line in combination with monoclonal antibodies (358, 367).

To avoid NK cell exhaustion, targeting cytokine-related immune checkpoints is another interesting approach. Several proteins including Suppressors of cytokine signaling (SOCS1–7 and CIS) downregulate cytokine signaling *via* JAK/STAT pathway in NK cells. These receptors are induced by cytokines such as IL-2 and IL-15 (368, 369) which are commonly used in NK *in vitro* expansion methods as it has been mentioned above, and consequently, their interruption could increase activity and persistence of NK cells. Studies from different groups have shown that the ablation of CISH in NK cells increased their cytotoxic properties (357) and even improved their metabolic fitness (94). In the context of CAR-NK cells, Rezvani’s group also showed an enhanced expansion capacity and cytotoxicity against leukemic cell lines when CD19 CAR-NK cells co-expressing hIL15 were used and demonstrated that this effect is in part related to an increase in the metabolic activity of CAR-NK cells (93). In a similar way, targeting SOCS3, another suppressor of cytokine signaling, resulted in NK cells with higher proliferation capacity and antitumor capacity (370). Another cytokine-induced intracellular checkpoint that has been targeted in NK cells is CLBL (Casitas B-lineage lymphoma pro-oncogene-b), an E3 ubiquitin ligase that mediates degradation of LAT protein, impairing the immunological synapse between NK cells and target cells (371). CLBL KO in placental-derived NK cells increased their cytotoxic potential against liquid tumors *in vivo* (372).

One of the greatest challenges in CAR therapy, especially in solid tumors, is to skip the immunosuppressive microenvironment generated around the tumor. Although many different cell types and molecules can contribute to this effect, as we previously discussed, TGF-β seems to be a key regulator of TME (373) and most of the efforts in this area have been directed to target this signaling pathway. Several groups have already successfully edited NK cells to disrupt the TGFβ-R2 (TGF-β receptor type 2) in NK and CAR-NK cells which conferred them resistance to TGF-β inhibition *in vitro* (374) and therefore enhanced tumor control against difficult to treat tumors such as prostate adenocarcinoma (375) or glioblastoma (376). Importantly, this study also showed that knocking out CD9 and CD103, surface ligands in NK cells interacting with αv Integrins, can inhibit TGF-β1 release by glioblastoma stem cells and increase NK cytotoxic effect (376).

Another drawback to consider in CAR-T and NK therapy is that in some cases tumor antigens targeted by CAR molecules or monoclonal antibodies are not restricted to malignant cells, but they are also expressed in the therapeutic NK cells, resulting in fratricide and consequently, in a decreased response to treatment due to death of effector cells. Elimination of the CAR-targeted receptor in CAR-NK cells is crucial to develop an effective immunotherapy product, particularly in the context of hematologic tumors. This is the case of CD70-CAR-NK cells that constitute a promising therapy for both solid and hematologic malignancies but during *in vitro* feeder cell-dependent expansion and activation, CD70 is upregulated in NK cells which results in fratricide. By eliminating CD70 in NK cells using CRISPR/Cas9, fratricide-resistant cells were obtained without affecting their cytotoxic potency (129). Following a similar strategy, Gurney et al. have demonstrated that CD38 knock-out by CRISPR/Cas9 in CD38-CAR-NK cells results in a decreased cell death due to fratricide and a more potent cytotoxic response of CAR-NK cells against AML primary cells (127). In MM, where CD38 is highly expressed in malignant plasma cells, daratumumab treatment together with a concomitant CD38 expression in NK cells leads to a marked decrease in NK cell numbers due to fratricide. Consequently, CD38 KO NK cells blocked daratumumab-induced fratricide, showing an improved metabolic profile and consequently enhanced cytotoxic activity against CD38 expressing MM cell lines and primary cells (377, 378). In this setting, it is worth mentioning that a clinical trial in MM is already ongoing to test FT576, iPSC-derived BCMA CAR NK cells in which CD38 has been ablated to avoid mAb-mediated fratricide, in combination with other drugs (NCT05182073). These results pave the way to optimize other CAR-NK cells that are being developed for BM malignancies and target antigens that are also expressed in NK cells such as the NKG2D-L MICA or CD7 (97, 146, 379) as previously shown in CAR-T cells (124, 125).

As it has been mentioned above chemokine signaling plays an important role in CAR-NK biodistribution and determines antitumor efficacy depending on tumor location. In this context, CRISPR gene editing can be applied to modulate NK cell trafficking. Levy et al. showed that CCR5 disruption using CRISPR/Cas9 modified NK cell migration *in vivo*, which reduced trafficking to the liver and increased BM homing (325). This approach seems promising to re-direct CAR-NK cells and increase their potency against BM-resident hematological malignancies. Similar knock-out and even knock-in strategies could be applied to target or express other chemokine receptors on the surface of CAR-NKs and redirect them to the tumor site. This strategy has been followed in NK-92 cells where overexpression of chemokine receptor CXC chemokine receptor 2 (CXCR2) and IL-2 by HDR mediated gene editing increased NK92 migration to tumor sites and improved tumor growth inhibition *in vivo* in a human colon cancer model (380).

### Future directions in CAR-NK cells genome-editing

One of the advantages of using CRISPR/Cas9 system in comparison with previously designed nucleases is the potential of this editing tool to target several loci at the same time. This greatly increases the possibility to optimize NK and CAR-NK cell cytotoxicity and persistence by multiplex targeting. Some examples include the combination of CISH and TGFBR2 targeting in NK cells (381) which increases cytotoxic activity against different hematological tumors. However, this type of strategies must be considered in detail since targeting several genes can also decrease NK survival and increase the risk of off-targets, including the risk of translocations (355, 356) as has been previously observed in CAR-T cells (382).

Thanks to the CRISPR/Cas9 system versatility, knock-in strategies focused on the delivery of a specific CAR molecule in a desired region of the genome can be implemented to increase the safety of the strategy. Although the efficiency of this strategy is still suboptimal in comparison with knock out approaches due to the intrinsic lower efficiency of HDR versus NHEJ (352) and the NK reluctance to the different donor template delivery systems previously mentioned, recent studies demonstrated the feasibility to deliver CAR molecules to NK cells using CRISPR/Cas9 tools, resulting in an increased killing capacity of the effector cells when an EGFR-CAR was used against prostatic adenocarcinoma (375) and a CD33-CAR in an AML model (383). This will also allow the combination of different modifications to improve CAR-NK functionality to target challenging tumor cell types.

The recent description of new gene-editing tools that do not generate double-strand breaks in the genome, such as Base editing (384, 385) and Prime editing (386) are a promising strategy for the multiplex gene-editing approaches in CAR-NK cells. In both cases wild type Cas9 is substituted by a catalytically impaired Cas9 protein (dead Cas9 or Cas9 nickase) that will not generate DSBs in the genome, minimizing the risk of potential off-targets or translocations and improving the safety of the therapeutic product.

## Conclusions

Over the last years, preclinical studies and preliminary clinical evidence indicate that “off-the-shelf” allogeneic CAR-NK therapy is a novel platform with a better safety profile than autologous CAR-T due to the low incidence of adverse events. Fast and high-quality responses achieved in a limited number of clinical trials with available results point to the efficacy of CAR-NK cell therapy in treating CD19^+^ relapsed/refractory tumors. Results of several ongoing clinical trials are awaited to clarify the broad applicability and long-term responses of CAR-NK in monotherapy or combinatorial approaches. Preclinical studies also anticipate the existence of CAR-independent challenges that hinder CAR-NK long-term function, leading to tumor resistance. Many approaches described in this review are under investigation to deeply understand these mechanisms and their involved targets, which differ between NK cell sources, triggering exhaustion and limited expansion and persistence *in vivo*. Although “optional” in allogeneic NK cells compared to allogeneic CAR-T therapy, CRISPR/Cas9 gene-editing constitutes a key strategy to obtain multi-engineered antitumor effectors to surpass these obstacles and outperform undesired effects of other combinatorial approaches, such as mAbs or inhibitors. Multiplex gene-edited CAR-NK products are exponentially growing in the field as an optimized “all in one” solution and have already become a clinical reality, offering an alternative to patients with limited treatment opportunities.

## Author contributions

All authors contributed to the article and approved the submitted version. AV and JM-L conceptualized and designed the manuscript. All authors were involved in the literature collection and writing. AV, AG-O, and EC designed and created the figures, and AV and EM-M generated the clinical trial table. All authors contributed to the article and approved the submitted version.

## Funding

This work was supported by the Research Institute Hospital 12 de Octubre (i+12) and by Instituto de Salud Carlos III (ISCIII)/Red Española de Terapias Avanzadas RICORS/TERAV -RD21/0017/0027 and RD21/0017/0030- Supported by European Union-NextGenerationEU. Plan de Recuperación Transformación y Resiliencia and grants PI18/01519 from Instituto de Salud Carlos III (ISCIII) and from the CRIS foundation to JM-L. AV and EM-M is supported by Research Institute Hospital 12 de Octubre (i+12). AG-O is supported by HIGEA 2019/0123 AIE project to JM-L. EC is supported by a Fellowship from Spanish Ministry of Science, Innovation and Universities (FPU18/02963). LC is supported by ICI20/00022 project to JM-L. JE is supported by a Fellowship from Instituto de Salud Carlos III (ISCIII) (IFI18/00034). AL is supported by Advanced Therapies National Network RICORS (Rd21/0017/0030).

## Conflict of interest

JM-L has received grant support from BMS; and has performed consultancy work for BMS, Janssen, Novartis, GSK, Incyte, Roche, and Astellas. PR has licensed medicinal products and receives research funding and equity from Rocket Pharmaceuticals, Inc., Patents&Royalties, Research Funding.

The remaining authors declare that the research was conducted in the absence of any commercial or financial relationships that could be construed as a potential conflict of interest.

## Publisher’s Note

All claims expressed in this article are solely those of the authors and do not necessarily represent those of their affiliated organizations, or those of the publisher, the editors and the reviewers. Any product that may be evaluated in this article, or claim that may be made by its manufacturer, is not guaranteed or endorsed by the publisher.
